# Understanding older adults’ smartphone addiction in the digital age: empirical evidence from China

**DOI:** 10.3389/fpubh.2023.1136494

**Published:** 2023-07-07

**Authors:** Yujing Xu, Kai Zeng, Lucong Dong, Xiaofen Zheng, Yuxiu Si

**Affiliations:** ^1^School of Management, Zhejiang University of Technology, Hangzhou, China; ^2^School of Economics, Zhejiang University of Technology, Hangzhou, China

**Keywords:** smartphone addiction, older adults, subject cognitive decline, self-control theory, alienation

## Abstract

**Background:**

Despite the fact that an increasing number of older adults are addicted to smartphones, the existing addiction literature still focuses primarily on adolescents. To address this issue, this study draws from the perspectives of subjective cognitive decline and family relationship conflict to examine older adults’ smartphone addiction based on their key characteristics.

**Methods:**

This study investigates the effects of subjective cognitive decline and family relationship conflict on older adults’ smartphone addiction through a survey of 371 subjects in China.

**Results:**

The results show that subjective cognitive decline and family relationship conflict affect older adults’ smartphone addiction through a sense of alienation. In addition, older adults’ perceived power moderates the relationship between alienation and smartphone addiction.

**Discussion:**

This study offers new perspectives on the study of smartphone addiction from the perspective of older adults, and sheds light on how to improve the older adults’ quality of life in their later years.

## Introduction

According to a recent report,^1^ at the end of 2021, the number of internet users aged 60 and above reached 119 million. With the popularity of smartphones, an increasing number of older adults have adopted and are using smartphones, which has become a general trend (1). It is worth noting that smartphone addiction may be present in older adults, as they may spend too much time on their smartphones (2). Smartphone addiction has been defined as the maladaptive dependence or compulsive use of smartphone devices (3). Smartphone addiction is often associated with excessive smartphone use (4), and overuse or misuse of personal electronic devices can reduce health-related quality of life and impair some abilities (5), especially in older adults. In an aging society, the base of the older adults has increased, and their misuse of mobile phones is widely spotted and recognized by scholars.

Although scholars have long focused on smartphone addiction (6, 7), current research on smartphone addiction has mainly focused on adolescents (8) and has ignored the problem of smartphone addiction in older adults (2). Older adults have had late access to the internet, and their attitudes toward it and the psychological states behind addiction differ from those of adolescents (9). This leads to the question of whether smartphone addiction among middle-aged and older adults has unique mechanisms, and the factors influencing have not been identified in the literature. In fact, the characteristics of older adults and the external environment in which they live are different from those of young people, resulting in both similarities and differences between their smartphone addiction and that of other age groups. Therefore, we consider the influence of both older adults characteristics and their external environments on their smartphone use behavior.

Subjective cognitive decline is a common problem characterized by memory loss, which is most unique and typical for older adults (10, 11). Subjective cognitive decline in older adults is closely associated with their self-control and compulsive use. In addition, the family is the most common environmental factor influencing people’s addictive behaviors and represents the environment in which the older adults are most frequently contacted. Conflict is universal in the family setting (12), and family relationship conflict depends on the frequency and intensity of interpersonal conflicts between family members in the family system (13). Conflict in family relationships can affect the psychological state and even the quality of life of older people (14). Therefore, we suggest that subjective cognitive decline and family relationship conflict are the factors with the greatest impact on smartphone addiction in older adults.

In addition, most older adults suffer from social disconnection (15), and the effect of social disconnection results in their alienation. Alienation is defined as a loss of connection with oneself and others and incidental negative feelings, typically exhibited as feelings of loneliness, uncontrollability, hopelessness, and so forth (16–18). The sense of alienation involves feeling of being alienated from society, nature, and their various relationship networks. Past research on alienation has focused more on adolescents, suggesting that alienation leads to adolescent smartphone addiction (19). The relationship between alienation and smartphone addiction in older adults has rarely been discussed. However, elders who feel alienated have unmet emotional needs and are more likely to seek solace in the virtual world (20). Therefore, they communicate less with others in reality, leading them to use their smartphones more, resulting in smartphone addiction (21, 22).

In this study, we attempted to reveal that older adults are susceptible to smartphone addiction in the era of digitalization. By exploring the causes of smartphone addiction among older adults in terms of subjective cognitive decline and family relationship conflict, we proposed that self-control deficits are crucial for older adults to become addicted to smartphones. In addition, we investigated the role of perceived power in moderating the older adults based on the approach-inhibition theory of power. To validate our model, we used a questionnaire survey to conduct an in-depth study of smartphone addiction among middle-aged and older adults. A total of 371 valid questionnaires were received. In the subsequent data analysis section, we used SmartPLS to conduct a simple validation of the model, demonstrating the effects of subjective cognitive decline and family relationship conflict on smartphone addiction among older adults. Thus, we provide valuable insights for research on smartphone addiction in the era of digitalization.

## Literature review

### Smartphone use of the older adults

According to existing studies, smart devices have played an important role in people’s lives, and many older adults have started to use smartphones for various needs. First, some older adults may be in poor health and have distant relationships with their children, and most live alone and need to communicate with their children and friends through smartphones to meet their emotional and health needs. Second, some older adults are restricted from going out due to the decline in their physiological functions (23), leading them to use smartphones because they have nothing to do and are lonely. Third, older adults need smartphones. When older adults, such as those who have Jitterbug phones, use their smartphones for entertainment, because the entertainment software is simple and does not require too much effort to use, it is easier for them to use and easily causes smartphone addiction. Nahas et al. (2) also warned that older adults use smartphones frequently, and such use requires continued investigation. According to the Internet Life of the Older Adults 2020 report, more than 100,000 older adults are online for more than 10 h a day. Older adults are fundamentally different from teenagers, both physically and psychologically. Therefore, the issue of smartphone addiction among middle-aged and older adults is worth studying, especially the relationship between physiological factors and smartphone addiction.

### Smartphone addiction of the older adults

Existing studies have proposed a number of terms to describe smartphone addiction. These terms include smartphone addiction (24), smartphone overuse (25), and problematic smartphone use (26). In the identified literature, there is currently no exact distinction among these terms (27), and most studies use the terms smartphone addiction and excessive smartphone interchangeably. Therefore, we do not differentiate between smartphone addiction and smartphone overuse and use the definition of smartphone addiction to describe the phenomenological problem of addiction to smartphones.

Smartphone addiction is often defined as an obligatory pattern of smartphone use (4). It is generally associated with adverse outcomes, such as reduced productivity, disrupted relationships (5), and anxiety (4). We reviewed the literature and found that smartphone use is an avoidance strategy to distract from negative emotions, which may lead to smartphone addiction (4). For example, if an individual suffers from anxiety, negative emotions may be resisted through entertainment, which can lead to smartphone addiction (28). Researchers have conducted numerous studies to explore the abovementioned problem, but most addiction research has focused on the psychological factors behind addiction (29), with few studies examining smartphone addiction from a physiological perspective, such as memory loss and reduced control. Berger, Wyss and Knoch (30) noted that control deficits can lead to smartphone addiction. Moreover, many existing factors can lead to control deficits, such as subjective cognitive decline (10). Therefore, the relationship between physiological factors and smartphone addiction deserves attention.

### Subjective cognitive decline

Subjective cognitive decline has received increasing attention from scholars (10). Subjective cognitive decline is self-experienced cognitive decline and can be caused by factors such as depression and anxiety (31) and varies across individuals (10). However, most cognitive abilities are in a stage of decline with age. Therefore, we believe that the problem of subjective cognitive decline is more severe in older adults than in younger adults. Early studies on subjective cognitive decline have emphasized memory decline (32), and more recent studies have found an association between subjective cognitive decline and other cognitive problems (10) that affect the executive control functions of the brain (33, 34). In the context of subjective cognitive decline in older adults, deficits in executive function due to memory loss and reduced control make them engage in problematic use behaviors. However, little literature has explored the factors associated with smartphone addiction in terms of subjective cognitive decline, and a framework model is needed to explain the link between the two.

### Self-control theory and executive control

The terms executive control and central executive are often used interchangeably. However, in a broad sense, executive function is a mental construct of the cognitive functions required for the conscious, top-down control of actions, thoughts, and emotions (35). These functions are associated with control and higher cognition. Impaired executive functions can lead to executive control deficits (36), and there is a strong relationship between them and behavioral problems (37). Based on existing behavioral addiction research, deficits in executive control functions are an important cause of addiction (36, 38). Deficits in executive control can lead to consequences such as impulsivity, diminished control, dysphoria, and reduced control over problem behaviors and cause smartphone addiction (39, 40). Although researchers have demonstrated the relationship between executive control and smartphone addiction, few studies have further investigated smartphone addiction with the help of self-control theory. The development of executive deficits is accompanied by decreased self-control, increased cravings for short-lived pleasure (41), and a greater than usual likelihood of deviant behavior. Therefore, it is necessary to further explore smartphone addiction through executive control based on self-control theory.

Self-control theory was proposed based on the classical view of human behavior. All human behavior can be understood as the self-interested pursuit of pleasure or the avoidance of pain. Based on this, most scholars have expanded on self-control. Metcalfe and Mischel (42) proposed a dual hot and cold self-control system. The “hot” system is a dynamic automatic system, and the “cold” system is an informational control system. When the “cold” system is insufficient, the “hot” system controls the individual’s behavior. In the case of impaired executive function in older adults, the “cold” system is impaired, and emotions influence behavior. According to self-control theory, decreased self-control leads to more deviant behavior (41), such as overreliance on smartphones (43). Numerous studies have confirmed that self-control has an impact on individuals’ emotions and their behaviors. For example, De Ridder et al. (44), based on a pooled sample of 666 to 12,870 participants, showed that self-control was consistently associated with deviant and addictive behaviors. Other studies have shown that self-control is essentially a part of executive control, and when executive control is faulty, self-control inevitably suffers (45). Therefore, individuals with impaired executive function have reduced self-control and are associated with an increased likelihood of smartphone addiction.

### Approach-inhibition theory of power

The approach-inhibition theory of power proposes that increased power activates behavioral tendencies associated with the approach, and decreased power activates behavioral tendencies associated with inhibition (46). Based on this, most scholars have conducted research on power. Power is a core motivator in society (47) and is defined as the ability to change the state of others by providing or withholding resources and imposing punishment. Both increases and decreases in power can impact individual behavior (48). Research has shown that high power motivates individuals to engage in proximity-related behaviors, and participants with a greater sense of power also perceive more social rewards and less socialization (46). Fewer social activities are associated with a sense of alienation; therefore, we believe it is necessary to examine the relationship between a sense of alienation and smartphone addiction in light of the approach-inhibition theory of power. In this study, we refer to the power felt by older adults as perceived power. According to the approach-inhibition theory of power, individuals with high perceived power are more inclined to make autonomous decisions and less likely to be disturbed by outside interference. Older adults with high perceived power experience negative emotions such as stress when they cannot control their children (49), further increasing the likelihood of smartphone addiction. Therefore, we believe that it is necessary to examine the role of perceived power in the relationship between alienation and smartphone addiction.

## Hypothesis development

Older adults are at high risk of subjective cognitive decline (11). The most characteristic feature of subjective cognitive decline is a decline in memory (50), which is usually accompanied by cognitive problems such as a decline in control (10). Decreased control results in the inability of older adults to perform behaviors that require high levels of control, i.e., executive control impairment (36). Executive control usually involves conscious behavior and processing (51). When executive control is reduced, older adults are less able to inhibit their pleasure demands (52) and are inclined to repeat actions that please themselves. Studies have shown that smartphone use can boost an individual’s positive mood (53). The subjective cognitive decline in older adults makes them unable to restrain their tendency to derive pleasure from smartphone use, leading to constant phone use and ultimately resulting in smartphone addiction (6). Other studies have shown that individuals with executive control deficits tend to do the same things repeatedly and unconsciously and have no control over the amount of time spent (54). In addition, older adults with executive control deficits cannot control how much time they spend using their smartphones, leading to smartphone addiction (38). Therefore, we suggest that when subjective cognition declines occur in older adults, executive control is compromised, affecting smartphone use behaviors and leading to smartphone addiction. Based on this, we propose the following hypothesis:

*H1*: The higher the degree of subjective cognitive decline is, the more likely it is that middle-aged and older adults become addicted to smartphones.

In families, relationship problems, such as conflicts among family members, are common (12). These family relationship problems can significantly impact the psychological status of older adults (14). When the level of family relationship conflict increases, the anxiety level of the older adults increases (55), and the bonds among family members weaken (56). Most middle-aged and older adults rely on their families for physical, social and economic support, and family is one of their main external links (14, 57). When their ties to their families weaken, they strengthen their relationships with others in other ways out of a need for social connection. Smartphones are used as one way to strengthen connections with others, and older adults can use their smartphones to meet their social needs (58). Anxiety created by the urgency to socialize can lead to a tendency to overuse smartphones, resulting in smartphone addiction (59). As middle-aged and older adults age, they experience psychological changes, and family relationship conflicts can leave them without adequate family support and cause them to feel bleak about their future lives (60). The older adults may divert negative emotions, for example, by using smartphones to alleviate dissatisfaction due to family (55). However, the probability of smartphone addiction increases if older adults are continuously satisfied with smartphones (56). Therefore, we believe that the stronger the family relationship conflict felt by elders is, the greater the likelihood of smartphone addiction. Based on this, we propose the following hypothesis:

*H2*: The more substantial the degree of family relationship conflict is, the more likely it is that older adults have smartphone addiction.

Older adults with a subjective cognitive decline also suffer from reduced executive functioning, slower reaction time (61), reduced ability to handle things (23), and inability to do many things in life independently. In this context, older adults are perceived as unable to cope with complex matters, have difficulty communicating effectively, and suffer alienation from the people around them (62). Their self-confidence can be undermined by the alienation of others and can cause them to retreat in social situations (15), making them reluctant to engage with people (63). The more older adults avoid social activities, the less connected they are to society, enhancing their senses of isolation (64) and alienation (62). Other studies have shown that older adults who suffer from subjective cognitive decline may suffer memory difficulties (65) and reduced total cognitive resources (23), which can affect their lives as they forget important persons and events, such as former friends and significant anniversaries. In such circumstances, the older adults perceive themselves as socially neglected and have more distant and unfamiliar relationships with others (66), further aggravating elders’ loneliness and thus enhancing their sense of alienation (62). Therefore, we believe that the more severe the subjective cognitive decline in older adults is, the stronger the sense of alienation generated (67). Based on this, we propose the following hypothesis:

*H3*: The higher the degree of subjective cognitive decline in older adults is, the stronger their sense of alienation.

The family is one of the most critical areas in the lives of the older adults (14, 68). In the context of family conflict, tensions between family members lead to progressive marginalization and weakened voices of the older adults in family relationships (69). The stronger the family conflict is, the stronger the feelings of being marginalized. Some elders in this situation may perceive themselves as somehow excluded from the family and treated differently by family members, resulting in a sense of alienation (70). Other studies have shown that strained interpersonal relationships can send individuals the message that everyone does not accept them, awakening feelings of rejection within the individual (71). When older adults suffer from strained family relationships, it can make them reject engaging in social behaviors with others, alienating them from society and creating a sense of alienation. In other words, in the case of conflicting family relationships, elders are not emotionally supported, which raises their sense of alienation. Therefore, we believe that the stronger the family relationship conflict among middle-aged and older adults is, the stronger the sense of alienation. Based on this, we propose the following hypothesis:

*H4*: The stronger the conflict in the family relationship is, the stronger the sense of alienation.

Most middle-aged and older adults feel some degree of alienation as they age (62), and they believe that they cannot integrate into society, creating negative emotions such as disappointment and anxiety, which affect their everyday lives. Older adults use smartphones as an avoidance strategy to mitigate the adverse effects of alienation on their lives (4). For example, older adults who experience emotions such as loneliness and anxiety may communicate with people through their smartphones or use entertainment to deal with their negative emotions, leading to the persistent use of their phones and resulting in smartphone addiction (53). Other studies have shown that elders who develop a sense of alienation mostly lack social connections, feel inadequately cared for and have emotional needs that are increasingly not met. As a channel of social communication, smartphones can satisfy the social and other emotional needs of the older adults in the absence of interpersonal interactions in reality (58), which can increase their frequency and willingness to use smartphones and lead to smartphone addiction (22, 72). That is, older adults spend much time using smartphones to alleviate their sense of alienation, resulting in smartphone addiction (21). Therefore, we believe that the higher the sense of alienation felt by elders is, the higher the likelihood of smartphone addiction among middle-aged and older adults. Based on this, we propose the following hypothesis:

*H5a*: The higher the sense of alienation felt by older adults is, the more likely it is that they have smartphone addiction.

*H5b*: A sense of alienation mediates the relationship between subjective cognitive decline and smartphone addiction.

*H5c*: A sense of alienation mediates family relationship conflicts and smartphone addiction.

As they age, older adults often crave power (73) to counteract the fear of facing death. According to power inhibition theory, higher power represents older adults prioritizing behaviors consistent with their goals (74). That is, the higher the perceived power of older adults is, the more likely it is that they engage in behaviors consistent with their needs when faced with the choice of alleviating alienation (75). With increased perceived power, older adults suffering from alienation are more eager to use smartphones and have a higher likelihood of smartphone addiction. Other studies have shown that having power motivates them to seek new social relationships. When the perceived power of older adults increases, they are motivated to seek new social relationships (76). Older adults who suffer from alienation lack social relationships, and perceived power exacerbates the likelihood that they will seek new social relationships. Once a response is received from a smartphone, it leads to continuous smartphone use, forming a habit of smartphone use and leading to smartphone addiction (77). Therefore, we suggest that the higher the perceived power of middle-aged and older adults is, the more likely it is that they will develop smartphone addiction from the sense of alienation. Based on this, we propose the following hypothesis:

*H6*: Perceived power positively moderates the relationship between alienation and smartphone addiction.

## Methods

### Data collection

The research topic of this study is smartphone addiction among older adults. To ensure the respondents’ appropriateness, an online questionnaire was used to conduct the survey study. In addition, to ensure the validity of the results of the empirical test part of this study, the questionnaire strictly adhered to the questionnaire design steps. First, taking into account the research context of smartphone use by older adults, the questionnaire was designed based on the literature, drew on mature variable measurement scales from reputable international journals and was translated into Chinese. Second, unsuitable items were modified appropriately according to the situation of older adults. Finally, the questionnaire was randomly distributed through Questionnaire Star, a professional website dedicated to data collection in the form of questionnaires in China. All measurements in this study used a five-point Likert scale.

The questionnaire was tailored to older adults aged 45 years or older to ensure the appropriateness of the respondents because the research context of this study was to explore the smartphone usage of older adults. As mentioned above, the participants had to be aged 45 or older and use a smartphone in their daily lives. There were no restrictions on gender, work or educational background. Individuals under the age of 45 were excluded, as were those who did not use a smartphone in their daily lives. People who answered the questionnaire too quickly, regularly or inconsistently were also considered invalid. A total of 408 replies were eventually submitted, and after removing elders under the age of 45, 371 valid surveys remained.

The sample size in this study is 371, which exceeds the planned sample size of 242. The planned sample size was calculated according to Gpower after determining the effect size, and the specific calculation process was as follows: First, Cohen suggested that an effect size f^2^ = 0.15 (equivalent to R^2^ = 0.13) is medium, so the planned effect size f^2^ was determined to be 0.15. In addition, to avoid low statistical power due to insufficient samples, the expected statistical power was set at 99% using strict criteria. The sampling plan of the study takes the main effect analysis as an example. If the estimated effect size f^2^ of the main effect is 0.15, the statistical power (1 − β) is 99%, and there are 4 control variables and 2 predictor variables, the sample size of the plan obtained by GPower 3.1 software is 242. Therefore, there is no problem of low statistical power due to insufficient sample size.

Among the final valid questionnaires, relatively more were filled out by men (*n* = 206, 55.53%); the average age was above 45 years old, with more people aged between 50 and 54 years old (*n* = 146, 39.35%); and the majority of the respondents had been using smartphones for more than 6 years (*n* = 214, 56.68%), as shown in Table 1 on the descriptive statistics.

**Table 1:** Descriptive statistics of respondents’ characteristics.

Demographics	Category	*n* = 371	Percentage (%)
Gender	Male	206	55.53
	Female	165	44.47
Age	45–49	119	32.06
	50–54	146	39.35
	55–59	72	19.41
	60–64	23	6.20
	Over 64	11	2.96
Education	Below primary school	3	0.81
	Primary school	4	1.08
	Middle school	27	7.23
	High school (or junior college, etc.)	85	22.91
	Bachelor’s degree (or college, etc.)	239	64.42
	Master’s degree	12	3.23
	Doctoral degree	1	0.27
Smartphone use time	Less than 1 year	6	1.62
	1–2 years	25	6.74
	2–4 years	51	13.75
	4–6 years	75	20.22
	More than 6 years	214	57.68

### Measure

We used well-established and repeatedly validated scales. These scales have been widely adopted by several scholars and have been proven to be credible. Since the items in this study’s scales were all derived from studies written in English and the respondents of this study’s questionnaire were older adults in China, we used the forward-backward translation method to translate the measurement items written in English. In addition, several elders were invited to check the understandability of the scale, and based on the feedback results, further adjustments and modifications were made. Based on feedback, the scale was further adjusted and modified to ensure its accuracy. The questionnaire consisted of 28 items in 5 constructs.

*Subjective cognitive decline* was assessed using a nine-item scale developed by Gifford et al. (78), which is a commonly used instrument to measure subjective cognitive decline in older adults. It measures the subjective perception of cognitive decline rather than objective cognitive decline or organic lesions. Sample items include “Do you think you have problems with your memory?” and “Do you have difficulty remembering a conversation from a few days ago?.” The Cronbach’s alpha of this scale was 0.91.

*Family relationship conflict* was assessed by means of a three-item scale adapted from Tepper et al. (79), which is a well-developed measurement with good validity and reliability. Sample items include “How much relationship tension is there between you and your family?” and “How much emotional conflict is there between you and your family?.” The Cronbach’s alpha of this scale was 0.79.

*Alienation* was assessed using an eight-item scale adapted from Banai and Reisel (80), which is a commonly used instrument to measure perceived feelings of being somehow abandoned. Examples are as follows: “I would prefer to live a different life than I do” and “Many people in our society are lonely and unrelated to their fellow human beings.” The Cronbach’s alpha of this scale was 0.81.

*Perceived power* was contextually adapted from two scales, sense of power and perceived influence, which were developed by Schaerer et al. (81) and Ju et al. (82). These two scales are brief and effective measures of power and influence over others, which are published in respected journals and used in many high-quality studies. We adapted the scales by selecting items from both scales that matched the characteristics of older adults. The adapted scale consists of four items, such as “I was able to induce a change in the actions of my child” and “I was able to control the actions of my child.” The Cronbach’s alpha of this scale was 0.78.

*Mobile phone addiction* was based on the scale of Kwon et al. (83). We combined the characteristics of the older people and integrated and reduced the items that did not fit their smartphone use and life situations. The adapted scale consists of four items, such as “Won’t be able to stand not having a smartphone” and “Feeling impatient and fretful when I am not holding my smartphone.” The Cronbach’s alpha of this scale was 0.81.

## Data analysis and results

**Figure 1:**
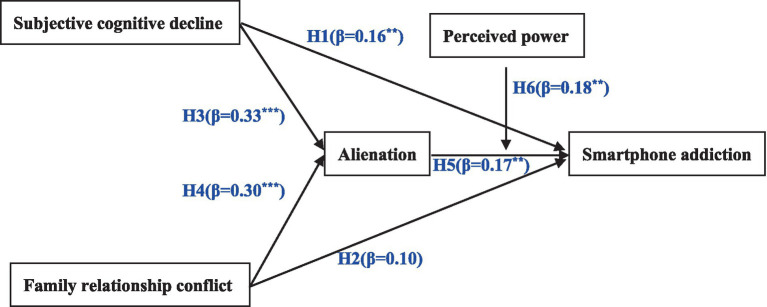
Research model.

A two-step analysis procedure was used to evaluate the research model (84). The measurement model was first examined, and then the structural model was assessed. First, Cronbach’s alpha (CA), composite reliability (CR), convergent validity, and discriminant validity were examined to check the reliability and validity of the survey instrument. Second, the research model was tested using partial least squares (PLS), a technique for modeling latent structural equations based on component evaluation. According to Banai and Reisel (80), our research model measures detachment in a second-order model. PLS is considered to be the appropriate choice for testing such a model. In addition, since we aimed to predict whether family relationship conflict and subjective cognitive decline affect smartphone addiction in older adults, the PLS method was applied to our research model (Figure 1). We used SmartPLS 3.0 software to test our model.

### Measurement model

CA and CR are typically used to test the consistency and stability of the scales. The reliability test results for this study were obtained by running the PLS algorithm in SmartPLS3.0, and the results are shown in Table 2. The CA values for all variables ranged from 0.78 to 0.91, and the CR values ranged from 0.84 to 0.93, which met the criterion of 0.70 (85). Table 2 indicates that this study has good reliability.

**Table 2:** Results of reliability, validity, and mean of the construct.

Variable	Item	Loading	Mean	S.D.	AVE	CA	CR
Subjective cognitive decline (SCD)	SCD1	0.81	2.94	0.90	0.58	0.91	0.93
	SCD2	0.79					
	SCD3	0.77					
	SCD4	0.73					
	SCD5	0.68					
	SCD6	0.73					
	SCD7	0.85					
	SCD8	0.69					
	SCD9	0.80					
Family relationship conflict (FRC)	FRC1	0.82	2.20	0.92	0.71	0.79	0.89
	FRC2	0.91					
	FRC3	0.79					
Alienation (Ali)	Ali1	0.78	3.14	0.74	0.58	0.81	0.86
	Ali2	0.79					
	Ali3	0.74					
	Ali4	0.72					
	Ali5	0.61					
	Ali6	0.84					
	Ali7	0.82					
	Ali8	0.77					
Perceived power (PP)	PP1	0.73	2.94	0.64	0.57	0.78	0.84
	PP2	0.90					
	PP3	0.65					
	PP4	0.74					
Smartphone addiction (SA)	SA1	0.84	3.12	0.75	0.64	0.81	0.87
	SA2	0.85					
	SA3	0.81					
	SA4	0.68					

AVE, Average variance extracted; CA, Cronbach’s alpha; CR, Composite reliability.

Convergent validity. In this study, convergent validity was mainly measured using indicator loading and average variance extracted (AVE). As shown in Table 2, the factor loadings of all of the variables were above 0.7 (86) and significant at the 0.001 level; all AVE values for all of the variables were above 0.5 (85). Therefore, this study has good convergent validity.

Discriminant validity. The Fornell-Larcker criterion was used to measure discriminant validity in this study. As shown in Table 3, the square root of AVE for all variables was greater than the correlation with other variables, indicating good discriminant validity (85).

**Table 3:** Correlations of the constructs.

	SCD	FRC	Ali	PP	SA
Subjective cognitive decline (SCD)	*0.76*				
Family relationship conflict (FRC)	0.17	*0.84*			
Alienation (Ali)	0.38	0.36	*0.66*		
Perceived power (PP)	−0.01	0.16	0.16	*0.76*	
Smartphone addiction (SA)	0.24	0.19	0.27	0.07	*0.80*

Italics are the square root of AVE.

In this study, Harman’s single factor method was used to include all of the variables in the study model in the factor analysis. The results showed that the maximum explanation rate of a single factor was 24.32%, below the 40% criterion (87), indicating that the issue of common method bias did not significantly affect this study.

### Structural model

In this study, SmartPLS 3.0 software was used, and the PLS algorithm was executed. Bootstrapping was run 5,000 times to assess the significance of the main effect path, and the results are shown in Table 4. The results show that the relationship between subjective cognitive decline and smartphone addiction was significant (*β* = 0.16, *p* < 0.01), and H1 was supported; the relationship between family relationship conflict and smartphone addiction was not significant (*β* = 0.10), and H2 was not supported; the relationship between subjective cognitive decline and alienation was significant (*β* = 0.33, *p* < 0.001), and H3 was supported; the relationship between family relationship conflict and detachment was significant (*β* = 0.30, *p* < 0.001), and H4 was supported; and the relationship between alienation and smartphone addiction was significant (*β* = 0.17, *p* < 0.01), and H5 was supported. In addition, we further examined the mediating role of alienation between subjective cognitive decline, family relationship conflict, and smartphone addiction. As shown in Table 5, it was confirmed that both subjective cognitive decline (*β* = 0.06, *p* < 0.01) and family relationship conflict (*β* = 0.05, *p* < 0.01) could have a significant effect on the smartphone use behavior of middle-aged and older adults through alienation, and hypothesis H5a and hypothesis H5b were supported. Moreover, the variance accounted for (VAF) ranged from 20% to 80%, indicating that alienation plays a partially mediating role.

**Table 4:** Results of the main effects test.

Hypothesis No.	Path	Path coefficient	Standard deviation	t-value	*p-*value	Does it support the hypothesis
H1	SCD → SA	0.16	0.06	2.60	0.01	Yes
H2	FRC → SA	0.10	0.06	1.81	0.07	No
H3	SCD → Ali	0.33	0.05	6.66	0.00	Yes
H4	FRC → Ali	0.30	0.05	6.39	0.00	Yes
H5a	Ali → SA	0.17	0.06	2.67	0.01	Yes

**Table 5:** Results of mediation effect test.

	Indirect effect	Total effect	*p*-value	VAF
SCD → Ali → SA	0.06	0.21	0.01	0.26
FRC → Ali → SA	0.05	0.15	0.01	0.33

VAF, Variance accounted for.

In this study, we used a two-stage PLS approach to verify the moderating effect (88). The results are shown in Table 6, based on which we can conclude that perceived power has a positive moderating effect on the relationship between alienation and smartphone addiction (*β* = 0.18, *p* < 0.01), i.e., hypothesis H6 is supported. In addition, we also tested for a moderating effect with mediation. The results are shown in Table 7, where the upper and lower limits of the bootstrap 95% confidence interval do not contain 0 in the high grouping section, indicating that perceived power can negatively influence addiction in older adults through a detachment-mediated effect.

**Table 6:** Moderating effect test of smartphone addiction.

Independent variable	Model 1	Model 2
Alienation (Ali)	0.17^**^	0.16^*^
Perceived power (PP)	0.04	0.02
Interactions		
Ali*PP		0.18^***^
R^2^	0.10	0.14
△R^2^	0.10	0.04

^***^p < 0.001; ^**^p < 0.01; ^*^p < 0.05. Ali*PP = The interaction term between Ali and PP.

**Table 7:** Results of moderated mediation test.

		Effect	BootSE	BootLLCI	BootULCI
SCD → Ali → SA	High	0.13	0.06	0.21	0.42
	Low	0.01	0.07	−0.08	0.19
FRC → Ali → SA	High	0.14	0.06	0.25	0.47
	Low	0.02	0.07	−0.04	0.23

High level is the mean plus one standard deviation, low level is the mean minus one standard deviation.

## Conclusion and discussion

In this study, we aimed to explore the effects of subjective cognitive decline and family relationship conflict on the addictive smartphone use of older adults. Moreover, to explain the reasons behind smartphone addiction more accurately and comprehensively, we explored the moderating role of perceived power on the relationship between alienation and smartphone addiction. The research hypotheses proposed were confirmed by combining the previous research results and the empirical findings in this study.

Based on the literature on older people’s situations and smartphone addiction, we developed and tested the literature linking older people’s characteristics to smartphone addiction (89–91). H1 of this study showed that subjective cognitive decline in older adults positively affected smartphone addiction with a path coefficient of 0.16, which is consistent with findings of altered usage behavior in the context of executive control deficits (54). The most typical characteristic of older adults with subjective cognitive decline is memory decline, which may be accompanied by impairments in control, leading to executive control deficits and thus an inability to control their use behaviors, resulting in smartphone addiction (92).

The most influential factor for older adults is family, apart from their idiosyncrasies (14). However, family relationship conflict has no direct effect on smartphone addiction, and H2 was not supported. One possible explanation is that when some elders experience family relationship conflict, they may be able to communicate with a third party other than family members to reconcile these intrafamily conflicts and mitigate the effects of family relationship conflict (93). Another possible explanation is that when some older adults experience family conflict, they convert this relationship into other emotions or feelings (60) that affect smartphone addiction behavior.

Studies have shown that declining subjective cognitive ability in older adults leads to memory loss, gradual detachment from those around them, and a gradual sense of not being cared for, which enhances their sense of loneliness (64), resulting in a sense of alienation (91, 94). This is consistent with our hypothesis. The results of H3 suggest that the path coefficient between subjective cognitive decline and alienation is 0.33. When subjective cognitive decline in middle-aged and older adults increases, alienation also increases.

We noticed that when family relationship conflict arises in elders, there is also a positive relationship with a sense of alienation. Family relationship conflict leaves the emotional needs of the older adults vacant and unmet, creating a sense of alienation (20). The results of hypothesis H4 prove this. Based on this, it is reasonable to suspect that alienation provides a bridge between family relationship conflict and smartphone addiction in older adults (91).

H5b proves the above conjecture. Although family relationship conflict cannot directly affect smartphone addiction among middle-aged and older adults, it can significantly affect older adults’ smartphone addictive behaviors through a sense of alienation. Older adults with family relationship conflicts are more likely to experience smartphone addiction when they feel alienated (93). Furthermore, our hypothesis is consistent with the results of H5 and H5a, where an increase in alienation positively affects smartphone addiction. The alienation generation in middle-aged and older adults can cause elders to develop negative emotions and try to eliminate alienation by other means, thus causing smartphone addiction (4). Therefore, subjective cognitive decline and family relationship conflict can positively influence smartphone addiction among middle-aged and older adults (95).

Regarding the influence of alienation on smartphone addiction, perceived control could affect their relationship in the context of smartphone use. Specifically, consistent with H6, perceived control may enhance the positive effect of alienation on smartphone addiction. Older adults, as they age and feel threatened by life, are more inclined to have the power to alleviate the anxiety and fear associated with death, which influences their usage behaviors (73, 90, 94). In this case, older adults have a deepened degree of perceived power and a more urgent need to escape a sense of alienation, which strengthens their smartphone use and is more likely to lead to smartphone addiction. Thus, perceived control positively moderates the relationship between alienation and smartphone addiction.

We demonstrated how subjective cognitive decline and family relationship conflicts contribute to smartphone addiction among middle-aged and older adults from the perspective of alienation (96, 97). Specifically, subjective cognitive decline and family relationship conflict reinforce older adults’ sense of alienation, thereby increasing the odds of smartphone addiction in middle-aged and older adults. The decline in subjective cognition in older adults is accompanied by a decline in control, resulting in executive control that can directly impact smartphone addiction. In addition, executive control positively moderates the relationship between alienation and smartphone addiction in the presence of high levels of perceived control and can deepen the effect of alienation on smartphone addiction.

### Theoretical implications

The present study sought to understand the impact of the unique characteristics of older adults on their smartphone addiction from the perspective of executive control and to construct a theoretical model for understanding this new phenomenon. The current findings suggest that all of the hypothesized associations are supported. The theoretical implications are as follows.

First, this study enriched the literature on smartphone addiction by providing a theoretical understanding of the causes of smartphone addiction in older adults. Most research has focused on smartphone addiction in adolescents (97), and relatively few studies on smartphone addiction in older adults have been conducted, especially on the mechanisms unique to this population (91). The study’s results show that subjective cognitive ability and the unique characteristics of older adults were significantly associated with alienation and smartphone addiction. Decreased subjective cognition is associated with executive functioning, which can affect subsequent psychological and behavioral outcomes and ultimately lead to some adverse outcomes (95). However, most studies have focused on the memory decline associated with subjective cognitive decline, ignoring the concept that decreased control can cause executive deficits.

Second, this study extended the application of self-control theory by examining smartphone addiction in middle-aged and older adults and further demonstrated the impact of deficits in executive function on use behavior. In past studies, although executive control has been considered related to smartphone addiction, few studies have elaborated on their relationship from the perspective of self-control theory. However, as individuals age, there may be impairments in executive control functions that further affect self-control (92). This study combined self-control theory and executive control based on research on smartphone addiction in middle-aged and older adults, which provided new ideas for subsequent research.

Third, this study linked the sense of power to smartphone use behavior, emphasizing the boundary effect of sense of power on smartphone addiction and providing a theoretical basis for more addiction research in the future. The critical role of sense of power for the older adults was demonstrated. Many scholars have subsequently investigated the relationship between middle-aged and older adults and their sense of power. We hypothesized that a sense of power affects the user behavior of older adults. The findings suggest that the stronger elders’ sense of power is, the more likely they are to cause smartphone addiction in the context of creating a sense of alienation.

### Practical implications

This study provided a new direction for product developers by examining the relationship between middle-aged and older adults and smartphone addiction and explaining how family relationship conflict and subjective cognitive decline affect older adults’ smartphone use behavior. Given the increasing number of products designed for the older adults in the age of global aging, how to fit the needs of the older adults has become one of the current market issues. The results of this study suggested that elders’ alienation influences their smartphone use behaviors. Based on the results of this study, developers can develop educational games, family interventions, apps, and even human-computer interaction therapy based on social emotional artificial intelligence (98–100) for middle-aged and older adults.

According to existing studies, older adults have more robust needs than teenagers regarding entertainment and other aspects of smartphone use. However, subjective cognitive decline is more likely to cause smartphone addiction, which can cause depression, anxiety and other psychological emotions and thus affect the quality of life and life satisfaction of older adults (101, 102). This study investigated the differences between elders and younger adults, analyzed the impact of their characteristics on smartphone addiction, researched smartphone addiction in elders, and proposed corresponding countermeasures to reduce the decline in quality of life in later life caused by smartphone addiction in elders (102). In addition, this study provided insights into the prevention of smartphone addiction among older adults and into the development of anti-addiction mechanisms.

Family relationship conflict is also a more prominent issue for elders, and a certain degree of family relationship conflict can promote deviant behaviors in elders. The effect of family relationship conflict on smartphone use addiction in older adults implies that smartphone addiction in older adults can be alleviated by improving family relationship conflicts. Furthermore, the positive effect of alienation on smartphone addiction can be further enhanced by perceived power. These findings have important theoretical and practical implications.

### Limitations and future research

Although we provided some new insights into smartphone addiction in older adults, there are limitations to our results for several reasons. First, there are few studies on smartphone addiction among middle-aged and older adults to date, and the definition and dimensions of smartphone addiction are ambiguous. We used one of the definitions of smartphone addiction, but there may be other types and definitions of addiction that could be distinguished in future studies. Second, the sample in this study was taken from within China and represents the experiences of only a limited group of people. Although the sample size is statistically sufficient to test the hypotheses, future studies should expand the sample size to produce stronger and more convincing results. Third, we focused on only the relationships among subjective cognitive decline, family relationship conflict, and smartphone addiction among older adults without considering other effects. Future research should focus on the effects of other psychological factors on smartphone addiction among older adults. Finally, the research on smartphone addiction in older adults discussed in this paper is gradually attracting attention, and new research is constantly emerging. Therefore, future research can systematically review the relevant literature using meta-analysis, which can effectively improve the quality of research.

## Data availability statement

The raw data supporting the conclusions of this article will be made available by the authors, without undue reservation.

## Ethics statement

The studies involving human participants were reviewed and approved by the School of Management, Zhejiang University of Technology. The patients/participants provided their written informed consent to participate in this study.

## Author contributions

YX contributed to the idea, conceptualization, resources, methodology, and drafting. KZ was responsible for the investigation, data collection, article supervision, and data curation. LD and XZ analyzed the data and drafted the main manuscript. XZ and YS contributed to the methodology, revision, and editing. All authors contributed to the article and approved the version submitted for publication.

## Funding

This study was funded by the Humanities and Social Sciences Foundation of the Ministry of Education of the People’s Republic of China (22YJC630175; 22YJAZH143), the Zhejiang Provincial Natural Science Foundation of China (LY22G010003), the Post-funded Project of National Social Science Foundation of China (22FGLB101), and the 2021 Zhejiang University of Technology Humanities and Social Sciences Pre-research Fund Project (SKY-ZX-20210198).

## Conflict of interest

The authors declare that the research was conducted in the absence of any commercial or financial relationships that could be construed as a potential conflict of interest.

## Publisher’s note

All claims expressed in this article are solely those of the authors and do not necessarily represent those of their affiliated organizations, or those of the publisher, the editors and the reviewers. Any product that may be evaluated in this article, or claim that may be made by its manufacturer, is not guaranteed or endorsed by the publisher.

## Footnotes

^1^China Internet Network Information Center.
